# iMethyl-Deep: N6 Methyladenosine Identification of Yeast Genome with Automatic Feature Extraction Technique by Using Deep Learning Algorithm

**DOI:** 10.3390/genes11050529

**Published:** 2020-05-09

**Authors:** Omid Mahmoudi, Abdul Wahab, Kil To Chong

**Affiliations:** 1 Department of Electronics and Information Engineering, Jeonbuk National University, Jeonju 54896, Korea; omidmahmoudi75@jbnu.ac.kr (O.M.); me.wahabqayyum@gmail.com (A.W.); 2 Advanced Electronics and Information Research Center, Jeonbuk National University, Jeonju 54896, Korea

**Keywords:** RNA N6-methyladenosine site, yeast genome, methylation, computational biology, deep learning, bioinformatics

## Abstract

One of the most common and well studied post-transcription modifications in RNAs is N6-methyladenosine (m6A) which has been involved with a wide range of biological processes. Over the past decades, N6-methyladenosine produced some positive consequences through the high-throughput laboratory techniques but still, these lab processes are time consuming and costly. Diverse computational methods have been proposed to identify m6A sites accurately. In this paper, we proposed a computational model named iMethyl-deep to identify m6A *Saccharomyces Cerevisiae* on two benchmark datasets M6A2614 and M6A6540 by using single nucleotide resolution to convert RNA sequence into a high quality feature representation. The iMethyl-deep obtained 89.19% and 87.44% of accuracy on M6A2614 and M6A6540 respectively which show that our proposed method outperforms the state-of-the-art predictors, at least 8.44%, 8.96%, 8.69% and 0.173 on M6A2614 and 15.47%, 28.52%, 25.54 and 0.5 on M6A6540 higher in terms of four metrics Sp, Sn, ACC and MCC respectively. Meanwhile, M6A6540 dataset never used to train a model.

## 1. Introduction

Presently, many possibilities of methylation as an additional post-transcriptional modification of RNA have been found in sequence RNAs particularly mRNA [1]. The first internal of the mRNA modification discovery is N6-methyladenosine (m6A) modification which plays a fundamental regulatory role in different biological processes, such as brain development abnormalities [2], mRNA stability and splicing [3], RNA localization and degradation [4] and microRNA biogenesis [5]. It was reported that m6A modification associated with lots of diseases such as thyroid tumor [6], prostate cancer [7], breast cancer [8,9,10], pancreatic cancer [11,12], leukemia [13] and etc. Undoubtedly, the identification of m6A sites would be a great benefit for cell biology and disease mechanism research.

The high-throughput laboratory techniques such as two-dimensional thin layer chromatography [14], high performance liquid chromatography [15] and next-generation sequencing techniques (e.g., m6A-seq [16] and MeRIP-Seq [2]) have been developed to identify m6A sites but all of these are time consuming and costly. Because of these restrictions of experimental methods, finding an accurate and fast computational method for m6A sites identification is a significant task.

To date, some computational methods [17,18,19] have been proposed to build a predictive model for detecting transcriptome and m6A sites in different species of RNAs such as *Saccharomyces cerevisiae*, *Homo sapiens*, *Mus musculus* and *Arabidopsis thaliana*. *S. cerevisiae* is one of the most widely utilized organisms in biotechnology over the globe. The first computational method was proposed by Schwartz et al., for identifying of m6A sites [20], where they used machine learning technique logistic regression and inputted handcrafted features.

Chen et al., developed two sequence based predictors for the detection of m6A sites in *S. cerevisiae* called iRNA-Methyl [17] and RAM-ESVM [18] by using the support vector machine through pseudo nucleotide composition and pseudo dinucleotide composition respectively. iRNA-Methyl and RAM-ESVM have an ability to predict with the accuracy of 65.59% and 78.35% respectively. Xing et al., also contributed to improve the efficiency for the identification of m6A sites by introduced RAM-NPPS [19] model in which they used position-specific condition propensity as feature representation by using support vector machine. Their contribution increased the accuracy of 79.59%. Last but not least, another model was built by the Leyi et al., called DeepM6APred [21] with the handcrafted features by using different machine learning and neural network techniques. Until now DeepM6APred is competing all the predictors by the accuracy of 80.50%. All of these methods were trained and tested by using Chen et al. dataset [17]. They used handcrafted features for the feature representation and machine learning algorithms for constructing the models. For the fair assessment of the performance, each model used 10 fold and jackknife cross-validation.

In this study, we aimed to construct a deep learning model on M6A2614 and M6A6540 datasets which were based on the pioneering work of Chen et al. [17] and Xiaolei Zhu et al. [20] respectively. The proposed predictor which is called iMethyl-deep has a novel and powerful method to identify m6A *S. Cerevisiae* sites by using single nucleotide resolution to convert RNA sequence into high-quality feature representation in the robust deep learning technique convolution neural network (CNN). It extracts the important features automatically from the inputted RNA samples. This idea purely implemented for multiple extents of features for which deep learning is more robust. The proposed model outperforms in comparison with the state-of-the-art methods and successfully achieves ACC of 89.19% and 87.44% on M6A2614 and M6A6540 benchmark datasets respectively.

## 2. Materials and Methods

### 2.1. Benchmark Datasets

Two benchmark datasets for the *S. cerevisiae* genome were used in this work. The first dataset, named M6A2614, was proposed by Schwartz et al. [22], contains 1307 positive RNA sequences as methylated sites and 1307 negative RNA sequences as non-methylated sites. Several state-of-the-art computational identifiers used the M6A2614 dataset for their predictors [17,18,19,21]. The second dataset is called as M6A6540 dataset which was introduced by Xiaolei Zhu et al.’s [20] contains 3270 positive RNA sequences regarded as methylated sites and 3270 negative RNA sequences regarded as non-methylated sites, all steps for preparing the dataset was mentioned in their work. Both M6A2614 and M6A6540 benchmark datasets are mutually exclusive and to avoid the redundancy both datasets used CD-HIT-EST software [23]. The length of each sequence is 51 bp in both benchmark datasets. A depiction of the datasets is shown in Table 1.

As per the literature, the datasets are divided into training and testing set. The training dataset is characteristically used for the learning of the model, whereas the testing dataset is worked to evaluate the model. The most effective way for testing is the *k*-fold cross-validation test [24], which we got the combinations of different independent test datasets.

### 2.2. Formulation and Representation of RNA Samples

It is important to make data in the form of deep learning recognition because all algorithms take input as a vector or discrete, so we formulated RNA sequences into vector form. It also needs to consider the loss of pattern sequence information while converting into vector form, mostly it happens in the discrete model. There are many introduced techniques to avoid it, for example, PseAAC [25], which is widely used in proteomics. There is some vigorous software regarding PseAAC known as PseAAC-Builder [26], Propy [27], and PseAAC-General [28] was developed as an open source. Another approach, Pseudo K-tuple nucleotide composition (PseKNC), was introduced to provoke different feature vectors for RNA and DNA sequences, which used widely in many research works [29,30,31,32]. The sequence of RNA in the benchmark datasets is represented as R={N1,N2,N3,N4…,Ni}, where N1 denoted as the first single nucleotide in a sequence, N2 the second nucleotide and so on until the end of the sequence. In each sequence, there are four nucleotides A,C,G,U represented as a string form with different combinations like AGCUAUAG…UGACAU.

We started with a suitable format of deep learning to convert an RNA sequence into vector form for the formulation of the sequence instead of manually crafted features such as chemical properties and nucleotide frequency. One-hot encoding is used for this purpose, which maps the categorical variables into a binary representation. The four unique nucleotides A, C, G, and U mapped as (1, 0, 0, 0), (0, 1, 0, 0), (0, 0, 1, 0), (0, 0, 0, 1) respectively. Several deep learning models used one-hot encoding for the representation of the sequences such as [33,34]. Each sequence in both datasets is 51 bp long and after one-hot encoding, it transformed into a matrix. The matrix is represented as 4 columns and 51 rows, each column signifies an RNA base of sequence and the rows signify mapped representations of unique nucleotides.

## 3. The Proposed Model

We presented a model based on a CNN instead of handcrafted features extraction models as a classifier such as support-vector machine (SVM) [17,35,36,37]. CNN has been used in deep learning techniques and the area of bioinformatics extensively [33,34,38,39,40] and also in other fields [41,42]. It has the ability to gather all the worthwhile features automatically from the RNA m6A sequences during the training process. The input of the iMethyl-deep is one-hot encoded RNA sequences, each one has a length of 51 bp and four channels. CNN is processed with various layers and functions such as the convolution layer, pooling layer, activation function, and dropout to get exceptional results. We implemented a grid search algorithm while the learning process of the model with different hyper-parameters tuning. The fine-tuning parameters consist of convolution layers, filters, filter size, pool-size, stride length, and dropout values. The range of hyper-parameters is illustrated in Table 2.

The best resultant optimized parameters were chosen while considering the minimum validation loss to evade the overfitting and underfitting. In the proposed model, we implemented two 1-D (one-dimensional) convolution layers, which are represented as Conv1D. Each layer of Conv1D has 16 filters, with a filter-size of five. However, the convolution layer has the most pivot functionality on CNN. It extracts the features from the RNA positive and negative samples of m6A sites. We used the L2 regularization and bias regularization as a parameter in the convolution layer to avoid the overfitting problem with the value of 0.001 for both Conv1D. The exponential linear unit (ELU) is used as an activation function. A group normalization layer (GN) was used after both convolution layers, which helped to decrease the outcomes of convolution layers produced by each filter of Conv1D. Group normalization distributes the outcomes of convolution layers into groups and performs the normalization in each group. The group size is set as four. After each GN layer, a max-pooling layer was implemented to reduce the redundancy of the features from preceding layers. We set the pool-size of 4 and stride of two in both layers. The dropout layer was used after the second max-pooling layer with a rate of 0.35, which prevents overfitting and enhances the authenticity of the model. The dropout layer works as a strainer to discard some intermediary features while the training period, by arbitrarily shutting down some neurons and setting zero value for them. We used flatten function to unstack all multidimensional tensors of previous layers into a 1D tensor and fed to the fully connected (FC) layer. FC layer has 32 hidden units and also uses the L2 regularization parameter for the weights and bias with the value of 0.0001. We used the ELU activation function for the FC layer. In the end, a fully connected layer was implemented with the sigmoid function for binary classification. Sigmoid function squeezes the output values between 0 and 1.

**Figure 1:**
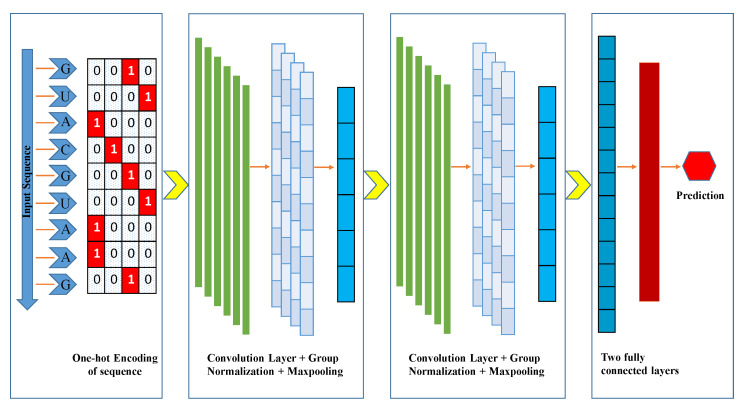
A graphical illustration of iMethyl-deep. Inputted RNA sequence converted into one-hot encoded, then fed into the Convolution Neural Network (CNN) layers for training the datasets.

The architecture of the proposed model is described in Table 3, where Conv1D (f, k, s) is a convolution layer as one-dimensional, parameter f is the number of filters, k is the kernel-size, and s represents the stride. ELU signifies as an activation function. The GroupNormalization (g) is a normalization layer, where g is a number of groups. The Maxpooling1D (l, r) is a max-pooling layer with two parameters, l is used as pool-size and r for the stride. The Dropout (d) denotes as a dropout layer with the value of d and the Dense (e) is a FC layer with the number of e nodes. At the last, the Sigmoid () function as an activation function makes it possible that the range of output should be between 0 and 1. Figure 1 demonstrates the comprehensive graphical architecture of the proposed model.

In iMethyl-deep, we used stochastic gradient descent (SGD) optimizer with the momentum of 0.95 and binary cross-entropy as a loss function [43], Learning rate for SGD is set as 0.003. The epoch and batch sizes are set to 100 and 32 respectively. The callbacks function is used to handle the checkpoint for saving the models and their best weights which have high accuracy. The early stopping is also used to stop the prediction accuracy when the validation stops improving, the value for the patience level is set to 30. The iMethyl-deep is implemented on the Keras framework [44].

## 4. Performance Evaluation

To calculate the performance of the prediction system, we used 10 folds cross-validation. Choosing a precise cross-validation method is a foremost part of investigating a prediction achievement. The *k*-fold cross validation method is a resampling method that provides a more accurate estimate of algorithm performance. It does this by first shuffling whole data and splitting them into *k* groups. Then the algorithm is trained and evaluated *k* times and the performance summarized by taking the mean performance score. Each unique group holds out as eight folds for training, one fold for validation, and the last one for testing. Each model was fitted on the training set and will be saved which one gives the highest accuracy on the validation fold. The performance of the model was evaluated on test fold, keeping the evaluated scores and abandoning the model. The Average scores of 10 repetitions were calculated and used as the performance evaluation of the proposed model. Four standard evaluation metrics were used in many research publication [45,46], which consist of overall accuracy (ACC), Mathew’s correlation coefficient (MCC), specificity (Sp), and sensitivity (Sn). The following are the mathematical formulation of four metrics [47,48,49,50].

(1) $$ACC=\frac{TP+TN}{TP+TN+FP+FN}$$

(2) $$SN=\frac{TP}{TP+FN}$$

(3) $$SP=\frac{TN}{TN+FP}$$

(4) $$MCC=\frac{TP\times TN-FP\times FN}{\sqrt{\left(TP+FP)\times (TP+FN)\times (TN+FP)\times (TN+FN\right)}}$$

where *TP* indicates a true positive which means a positive number of sequences predicted correctly and *TN* indicates as a true negative which can be described as a negative number of sequences predicted correctly. Meanwhile, *FP* designates as false positive which can be explained as a negative number of sequences identified falsely as positive and *FN* represents a false negative which means a positive number of sequences predicted falsely as negative. The receiver operating characteristics curve (ROC) and area under the ROC curve (AUC) are also used to evaluate the performance of the proposed model.

## 5. Results and Discussion

We evaluated the identification performance of our model, iMethyl-deep, on two RNA m6A benchmark datasets M6A2146 [22] and M6A6540 [20] for the *S. cerevisiae* genome. The results of the proposed model on the benchmark datasets show better performance in terms of all evaluation metrics. We used the same proposed model for both datasets.

### 5.1. The Performance of iMethyl-Deep on M6A2146 Benchmark Dataset

**Figure 2:**
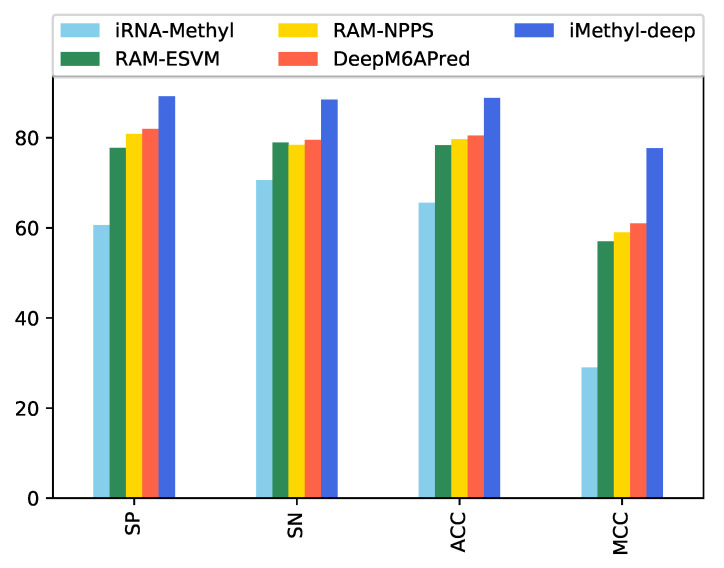
Performance evaluation illustration of iMethyl-deep on M6A2146 dataset.

**Figure 3:**
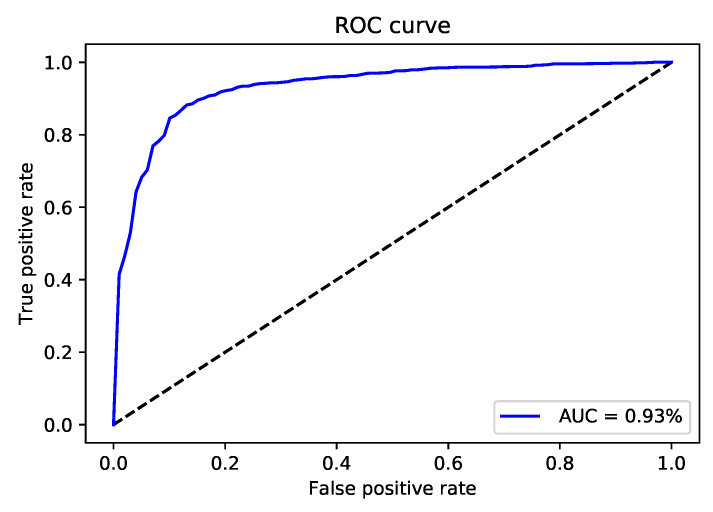
The receiver operating characteristics (ROC) curve of iMethyl-deep on M6A2614 dataset.

**Figure 4:**
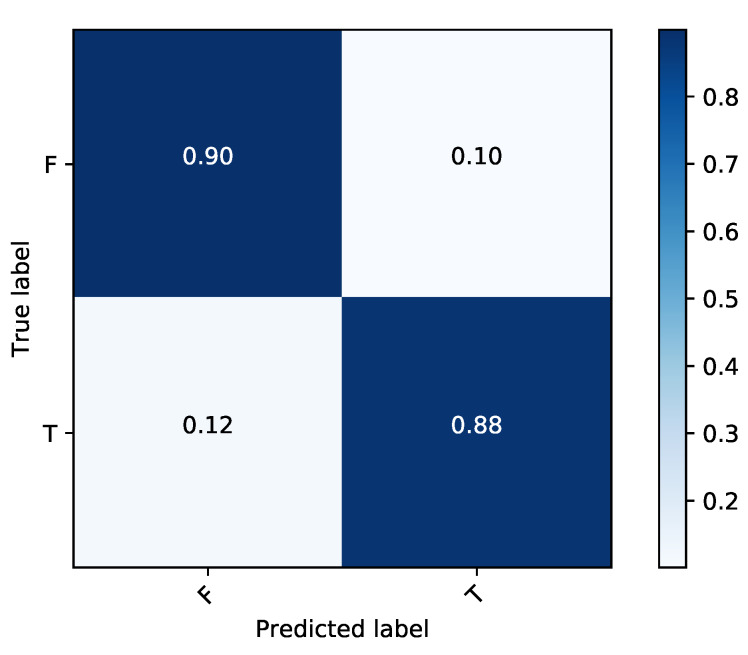
Graphical illustration of confusion matrix of iMethyl-deep on M6A2614 dataset.

After validating the effectiveness of the proposed method, by comparing its performance with four state-of-the-art methods iRNA-Methyl [17], RAM-ESVM [18], RAM-NPPS [19], and DeepM6APred [21] which used the same benchmark dataset, we obtained 89.92%, 88.46%, 89.19% and 0.783 for Sp, Sn, ACC and MCC respectively. Comparing with Deepm6Apred method, which is the best among the other existing methods, the performance of the proposed predictor is 8.96%, 8.44%, 8.69% and 0.173 higher in terms of four metrics respectively. We observed the proposed method is capable to distinguish m6A sites from non-m6A sites more accurately as compared to the other state-of-the-art predictors. Additionally, the less false positives are achieved by the highest Sp, which we reached. Table 4 shows the detail results of the iMethyl-deep model and Figure 2 represents the graphical illustration of results. We achieved 0.931 of AUC to prove the successful performance of the iMethyl-deep as depicted in Figure 3. The visualization representation of the confusion matrix is also shown in Figure 4.

### 5.2. The Performance of iMethyl-Deep on M6A6540 Benchmark Dataset

**Figure 5:**
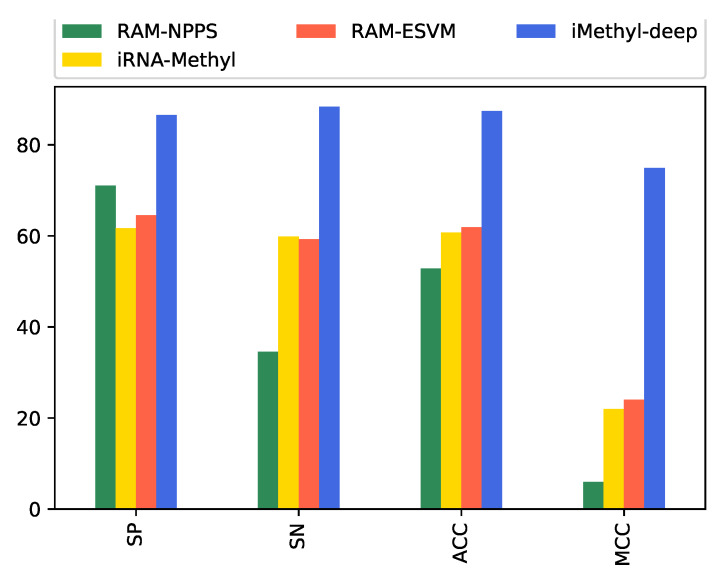
Performance evaluation illustration of iMethyl-deep on M6A6540 dataset.

**Figure 6:**
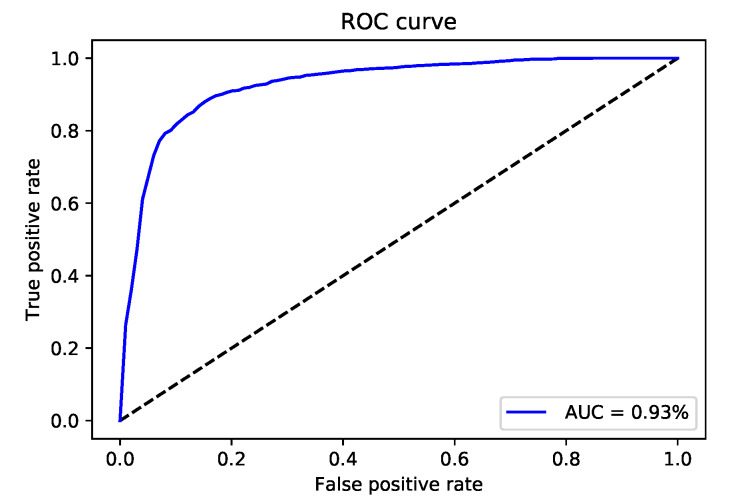
The receiver operating characteristics (ROC) curve of iMethyl-deep on M6A6540 dataset.

**Figure 7:**
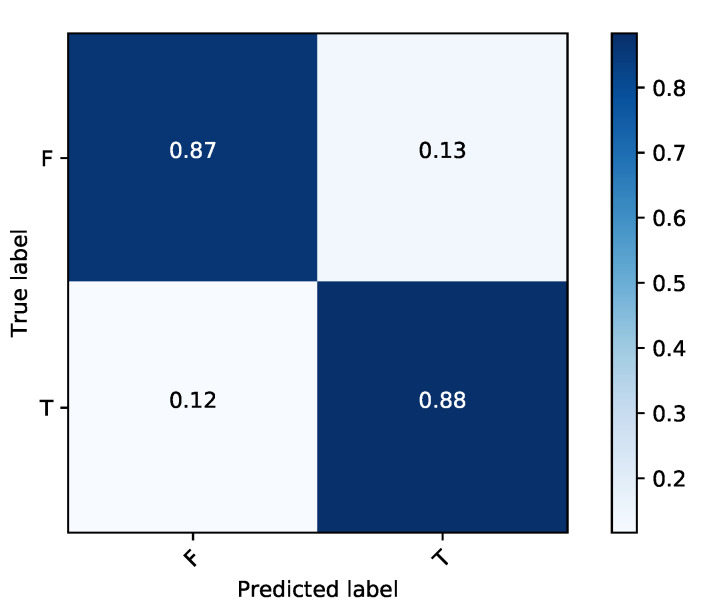
Graphical illustration of confusion matrix of iMethyl-deep on M6A6540 dataset.

In this section, the results of iMethyl-deep on benchmark dataset M6A6540 which were introduced by Zhu et al. [20] are shown. We should mentioned the DeepM6APred was just trained and tested on M6A2614 and not considered on M6A6540 dataset. Meanwhile, The M6A6540 never used to train in the other mentioned models. As shown in Table 5 and Figure 5, we obtained 86.54% of specificity, 88.34% of sensitivity, 87.44% of accuracy, and 0.749 of MCC. It is clear that our proposed model can outperform all four metrics in comparison with three state-of-the-art model RAM-NPPS [19], iRNA-Methyl [17] and RAM-ESVM [18] which had the maximum value for Sp, Sn, ACC and MCC repectively. Moreover, same M6A2146 dataset we reached to 0.931 of AUC for M6A6540 dataset. The AUC curve and the visualization representation of the confusion matrix are depicted in Figure 6 and Figure 7 respectively.

## 6. Conclusions

In this study, we proposed iMethyl-deep as a new computational predictor to identify N6-methyladenosine sites from RNA sequences. Two different benchmark datasets M6A2146 and M6A6540 were compiled to evaluate the performance of the proposed model. We used a one-hot encoding method to input RNA sequence and fed into a CNN. The simulated results show that iMethyl-deep can significantly and robustly improve the performance of deep learning to identify m6A sites. To access the effectiveness of the proposed predictor, we compared its performance with four state-of-the-art models. It predicts all evaluation metrics Sp, Sn, ACC, MCC and AUC better than the others. Potentially, the method proposed in this paper can be extended to be effective in brain development abnormalities, mRNA stability and splicing. In the future, we will further study in other kinds of modifications. The datasets and model is available at https://github.com/abdul-bioinfo/iMethyl-deep.

## Figures and Tables

**Table 1:** Benchmark datasets demonstration.

Datasets	Positive	Negative	Total
M6A2614	1307	1307	2614
M6A6540	3270	3270	6540

**Table 2:** Range of Hyper-parameters.

Parameters	Range
Convolution layers	[1, 2, 3, 4]
Filters in convolution Layer	[6, 8, 16, 24, 32, 44, 64]
Filter size	[2, 4, 5, 7, 8, 10, 13]
Pool-size in Maxpooling	[2, 4]
Stride length in Maxpooling	[2, 4]
Dropout values	[0.3, 0.35, 0.4, 0.45, 0.5]

**Table 3:** The architecture of the proposed model.

Layer	Output Shape
Input	(51, 4)
Conv1D(16, 5, 1)	(47, 16)
ELU	(47, 16)
GroupNormalization(4)	(47, 16)
MaxPool1D (4, 2)	(22, 16)
Conv1D(16, 5, 1)	(18,16)
ELU	(18, 16)
GroupNormalization(4)	(18, 16)
MaxPool1D(4,2)	(8, 16)
Flatten	(128)
Dropout(0.35)	(128)
Dense(32)	(32)
Dense(1)	1
Sigmoid	1

**Table 4:** Performance comparison of iMethyl-deep with other four state-of-the-art methods on M6A2614 dataset. Overall accuracy (ACC), Mathew’s correlation coefficient (MCC), specificity (Sp), and sensitivity (Sn).

Model	Sp (%)	Sn (%)	ACC (%)	MCC
iRNA-Methyl	60.63	70.55	65.59	0.29
RAM-ESVM	77.78	78.93	78.35	0.57
RAM-NPPS	80.87	78.42	79.65	0.59
DeepM6APred	81.48	79.50	80.50	0.61
iMethyl-deep	89.92	88.46	89.19	0.78

**Table 5:** The results of iMethyl-deep on benckmark M6A6540 dataset.

Model	Sp (%)	Sn (%)	ACC (%)	MCC
RAM-NPPS	71.07	34.59	52.83	0.06
iRNA-Methyl	61.68	59.82	60.75	0.22
RAM-ESVM	64.53	59.27	61.90	0.24
iMethyl-deep	86.54	88.34	87.44	0.74

## References

[1] Desrosiers R., Friderici K., Rottman F. (1974). Identification of methylated nucleosides in messenger RNA from Novikoff hepatoma cells. Proc. Natl. Acad. Sci. USA.

[2] Meyer K.D., Saletore Y., Zumbo P., Elemento O., Mason C.E., Jaffrey S.R. (2012). Comprehensive analysis of mRNA methylation reveals enrichment in 3 UTRs and near stop codons. Cell.

[3] Nilsen T.W. (2014). Internal mRNA methylation finally finds functions. Science.

[4] Meyer K.D., Jaffrey S.R. (2014). The dynamic epitranscriptome: N 6-methyladenosine and gene expression control. Nat. Rev. Mol. Cell Biol..

[5] Alarcón C.R., Lee H., Goodarzi H., Halberg N., Tavazoie S.F. (2015). N 6-methyladenosine marks primary microRNAs for processing. Nature.

[6] Heiliger K.J., Hess J., Vitagliano D., Salerno P., Braselmann H., Salvatore G., Ugolini C., Summerer I., Bogdanova T., Unger K. (2012). Novel candidate genes of thyroid tumourigenesis identified in Trk-T1 transgenic mice. Endocr. Relat. Cancer.

[7] Machiela M.J., Lindström S., Allen N.E., Haiman C.A., Albanes D., Barricarte A., Berndt S.I., Bueno-de Mesquita H.B., Chanock S., Gaziano J.M. (2012). Association of type 2 diabetes susceptibility variants with advanced prostate cancer risk in the Breast and Prostate Cancer Cohort Consortium. Am. J. Epidemiol..

[8] Akilzhanova A., Nurkina Z., Momynaliev K., Ramanculov E., Zhumadilov Z., Rakhypbekov T., Hayashida N., Nakashima M., Takamura N. (2013). Genetic profile and determinants of homocysteine levels in Kazakhstan patients with breast cancer. Anticancer Res..

[9] Reddy S., Sadim M., Li J., Yi N., Agarwal S., Mantzoros C., Kaklamani V. (2013). Clinical and genetic predictors of weight gain in patients diagnosed with breast cancer. Br. J. Cancer.

[10] Long J., Zhang B., Signorello L.B., Cai Q., Deming-Halverson S., Shrubsole M.J., Sanderson M., Dennis J., Michailiou K., Easton D.F. (2013). Evaluating genome-wide association study-identified breast cancer risk variants in African-American women. PLoS ONE.

[11] Lin Y., Ueda J., Yagyu K., Ishii H., Ueno M., Egawa N., Nakao H., Mori M., Matsuo K., Kikuchi S. (2013). Association between variations in the fat mass and obesity-associated gene and pancreatic cancer risk: A case–control study in Japan. BMC Cancer.

[12] Pierce B.L., Austin M.A., Ahsan H. (2011). Association study of type 2 diabetes genetic susceptibility variants and risk of pancreatic cancer: An analysis of PanScan-I data. Cancer Causes Control.

[13] Casalegno-Garduno R., Schmitt A., Wang X., Xu X., Schmitt M. (2010). Wilms’ Tumor 1 as A Novel Target for Immunotherapy of Leukemia.

[14] Keith G. (1995). Mobilities of modified ribonucleotides on two-dimensional cellulose thin-layer chromatography. Biochimie.

[15] Zheng G., Dahl J.A., Niu Y., Fedorcsak P., Huang C.M., Li C.J., Vågbø C.B., Shi Y., Wang W.L., Song S.H. (2013). ALKBH5 is a mammalian RNA demethylase that impacts RNA metabolism and mouse fertility. Mol. Cell.

[16] Dominissini D., Moshitch-Moshkovitz S., Schwartz S., Salmon-Divon M., Ungar L., Osenberg S., Cesarkas K., Jacob-Hirsch J., Amariglio N., Kupiec M. (2012). Topology of the human and mouse m6A RNA methylomes revealed by m 6 A-seq. Nature.

[17] Chen W., Feng P., Ding H., Lin H., Chou K.C. (2015). iRNA-Methyl: Identifying N6-methyladenosine sites using pseudo nucleotide composition. Anal. Biochem..

[18] Chen W., Xing P., Zou Q. (2017). Detecting N 6-methyladenosine sites from RNA transcriptomes using ensemble Support Vector Machines. Sci. Rep..

[19] Xing P., Su R., Guo F., Wei L. (2017). Identifying N 6-methyladenosine sites using multi-interval nucleotide pair position specificity and support vector machine. Sci. Rep..

[20] Zhu X., He J., Zhao S., Tao W., Xiong Y., Bi S. (2019). A comprehensive comparison and analysis of computational predictors for RNA N6-methyladenosine sites of *Saccharomyces cerevisiae*. Briefings Funct. Genomics.

[21] Wei L., Su R., Wang B., Li X., Zou Q., Gao X. (2019). Integration of deep feature representations and handcrafted features to improve the prediction of N6-methyladenosine sites. Neurocomputing.

[22] Schwartz S., Agarwala S.D., Mumbach M.R., Jovanovic M., Mertins P., Shishkin A., Tabach Y., Mikkelsen T.S., Satija R., Ruvkun G. (2013). High-resolution mapping reveals a conserved, widespread, dynamic mRNA methylation program in yeast meiosis. Cell.

[23] Fu L., Niu B., Zhu Z., Wu S., Li W. (2012). CD-HIT: Accelerated for clustering the next-generation sequencing data. Bioinformatics.

[24] Chou K.C., Zhang C.T. (1995). Prediction of protein structural classes. Crit. Rev. Biochem. Mol. Biol..

[25] Chou K.C. (2005). Using amphiphilic pseudo amino acid composition to predict enzyme subfamily classes. Bioinformatics.

[26] Du P., Wang X., Xu C., Gao Y. (2012). PseAAC-Builder: A cross-platform stand-alone program for generating various special Chou’s pseudo-amino acid compositions. Anal. Biochem..

[27] Cao D.S., Xu Q.S., Liang Y.Z. (2013). propy: A tool to generate various modes of Chou’s PseAAC. Bioinformatics.

[28] Du P., Gu S., Jiao Y. (2014). PseAAC-General: Fast building various modes of general form of Chou’s pseudo-amino acid composition for large-scale protein datasets. Int. J. Mol. Sci..

[29] Chen W., Lei T.Y., Jin D.C., Lin H., Chou K.C. (2014). PseKNC: A flexible web server for generating pseudo K-tuple nucleotide composition. Anal. Biochem..

[30] Chen W., Lin H., Chou K.C. (2015). Pseudo nucleotide composition or PseKNC: An effective formulation for analyzing genomic sequences. Mol. BioSystems.

[31] Chen W., Tang H., Ye J., Lin H., Chou K.C. (2016). iRNA-PseU: Identifying RNA pseudouridine sites. Mol. Ther. Nucleic Acids.

[32] Liu B., Fang L., Long R., Lan X., Chou K.C. (2016). iEnhancer-2L: A two-layer predictor for identifying enhancers and their strength by pseudo k-tuple nucleotide composition. Bioinformatics.

[33] Wahab A., Ali S.D., Tayara H., Chong K.T. (2019). iIM-CNN: Intelligent identifier of 6mA sites on different species by using convolution neural network. IEEE Access.

[34] Yu H., Dai Z. (2019). SNNRice6mA: A deep learning method for predicting DNA N6-methyladenine sites in rice genome. Front. Genet..

[35] Chen W., Ding H., Zhou X., Lin H., Chou K.C. (2018). iRNA (m6A)-PseDNC: Identifying N6-methyladenosine sites using pseudo dinucleotide composition. Anal. Biochem..

[36] Zhou Y., Zeng P., Li Y.H., Zhang Z., Cui Q. (2016). SRAMP: Prediction of mammalian N6-methyladenosine (m6A) sites based on sequence-derived features. Nucleic Acids Res..

[37] Chen W., Feng P.M., Lin H., Chou K.C. (2013). iRSpot-PseDNC: Identify recombination spots with pseudo dinucleotide composition. Nucleic Acids Res..

[38] Tahir M., Tayara H., Chong K.T. (2019). iDNA6mA (5-step rule): Identification of DNA N6-methyladenine sites in the rice genome by intelligent computational model via Chou’s 5-step rule. Chemom. Intell. Lab. Syst..

[39] Tahir M., Tayara H., Chong K.T. (2019). iRNA-PseKNC (2methyl): Identify RNA 2’-O-methylation sites by convolution neural network and Chou’s pseudo components. J. Theor. Biol..

[40] Akbar S., Hayat M., Iqbal M., Tahir M. (2020). iRNA-PseTNC: Identification of RNA 5-methylcytosine sites using hybrid vector space of pseudo nucleotide composition. Front. Comput. Sci..

[41] Ilyas T., Khan A., Umraiz M., Kim H. (2020). SEEK: A Framework of Superpixel Learning with CNN Features for Unsupervised Segmentation. Electronics.

[42] Zhang K., Zuo W., Zhang L. (2018). FFDNet: Toward a fast and flexible solution for CNN-based image denoising. IEEE Trans. Image Process..

[43] De Boer P.T., Kroese D.P., Mannor S., Rubinstein R.Y. (2005). A tutorial on the cross-entropy method. Ann. Oper. Res..

[44] Chollet F. Keras: Deep Learning Library for Theano and Tensorflow. https://keras.Io/.

[45] Manavalan B., Basith S., Shin T.H., Lee D.Y., Wei L., Lee G. (2019). 4mCpred-EL: An ensemble learning framework for identification of DNA N4-Methylcytosine sites in the mouse genome. Cells.

[46] Liu Z., Xiao X., Yu D.J., Jia J., Qiu W.R., Chou K.C. (2016). pRNAm-PC: Predicting N6-methyladenosine sites in RNA sequences via physical–chemical properties. Anal. Biochem..

[47] Chen J., Liu H., Yang J., Chou K.C. (2007). Prediction of linear B-cell epitopes using amino acid pair antigenicity scale. Amino Acids.

[48] Chou K.C. (2001). Using subsite coupling to predict signal peptides. Protein Eng..

[49] Chou K.C. (2001). Prediction of signal peptides using scaled window. Peptides.

[50] Zeng F., Fang G., Yao L. (2020). A deep neural network for identifying DNA N4-methylcytosine sites. Front. Genet..

